# Commentary: PLGA nanoparticles as an efficient carrier in *Toxoplasma* GAP45: a more effective vaccine against acute toxoplasmosis than traditional ones

**DOI:** 10.3389/fimmu.2025.1663012

**Published:** 2025-08-27

**Authors:** Nathkapach Kaewpitoon Rattanapitoon, Patpicha Arunsan, Nav La, Khristine Laguador Sandoval, Schawanya Kaewpitoon Rattanapitoon

**Affiliations:** ^1^ Parasitic Disease Research, FMC Medical Center of Thailand, Nakhon Ratchasima, Thailand; ^2^ Faculty of Medicine, Vongchavalitkul University, Nakhon Ratchasima, Thailand; ^3^ Faculty of Pediatric and Medicine, International University, Phnom Penh, Cambodia; ^4^ School of Arts and Sciences, National University, Pasay City, Philippines

**Keywords:** toxoplasmosis, subunit vaccine, PLGA nanoparticles, TgGAP45, nanovaccine delivery

The recent study by Zhou et al. (2025) demonstrated the use of PLGA nanoparticles to deliver the TgGAP45 antigen as a subunit vaccine against *Toxoplasma gondii*. This commentary highlights the originality and immunological sophistication of their approach, particularly the elicitation of balanced Th1/Th2/Th17 responses and significant protection in a murine model. We contextualize these findings within the broader vaccine landscape, contrasting them with other nanoparticle and antigen strategies. Furthermore, we propose future research directions not discussed by the authors, including mucosal delivery routes, application in chronic infection models, and systems immunology profiling. These suggestions aim to maximize translational potential and address current gaps in toxoplasmosis vaccine development.

## 1 Commentary

The study by Zhou et al. (2025) marks a significant advance in the field of vaccine immunology by demonstrating the immunoprotective potential of TgGAP45-loaded poly(lactic-co-glycolic acid) (PLGA) nanoparticles in acute toxoplasmosis. In contrast to conventional vaccine formulations using Montanide-based emulsions, this work elegantly integrates molecular parasitology, nanotechnology, and immunological profiling to offer a promising strategy for vaccine enhancement.

This study addresses one of the long-standing challenges in toxoplasmosis vaccine development: the need for sustained antigen presentation without compromising safety. The authors convincingly demonstrate that TgGAP45-PLGA nanoparticles not only enhance both humoral and cellular immune responses—including mixed Th1/Th2 and IL-17-driven Th17 profiles—but also significantly reduce parasite burden in target organs (heart and spleen), surpassing oil-adjuvanted formulations. This is a particularly notable achievement considering previous attempts with subunit vaccines have struggled to achieve robust CD8^+^ T-cell activation or durable protection (1, 2).

The mechanistic depth of the study is exemplary. Flow cytometry reveals enhanced dendritic cell maturation (CD83^+^/CD86^+^, MHC-I/II expression), while ELISA and lymphocyte proliferation assays substantiate broad cytokine induction, notably IFN-γ and IL-17. These findings align with the necessity for both antigen-specific cytotoxic responses and mucosal immunity in *T. gondii* control (3).

Compared to earlier efforts using SAG1-based or GRA7-based vaccines (4, 5), TgGAP45 targets the glideosome—a conserved and essential structure for motility and invasion. This strategic antigen selection distinguishes the present study from SAG1-centric vaccines that fail to elicit effective responses against intracellular tachyzoites. Furthermore, PLGA nanoparticles exhibit superior storage stability and safety over liposomes or lipid nanoparticles, which are prone to oxidative degradation (6).

Despite its many strengths, the study leaves several compelling avenues for future exploration.

### 1.1 Chronic infection model

While this work focuses on acute toxoplasmosis, the bradyzoite stage is responsible for chronic infection and reactivation. Evaluating vaccine efficacy in models using ME49 or VEG strains would enhance translational relevance (7).

### 1.2 Mucosal immunity and oral delivery

Given *T. gondii* is primarily acquired via the gastrointestinal route, oral or intranasal administration of TgGAP45-PLGA could be transformative. PLGA particles are known to be amenable to mucosal delivery and could be formulated with mucoadhesive polymers to promote Peyer’s patch uptake (8).

### 1.3 Systems immunology profiling

Bulk or single-cell RNA sequencing of splenocytes or DCs could elucidate transcriptional programs underpinning the observed immune response, enabling biomarker discovery for correlates of protection.

### 1.4 Cross-species application

Evaluating this platform in target livestock species (e.g., ovine or porcine models) would address the need for agricultural biosecurity and zoonotic transmission control.

### 1.5 Antigen multiplexing

Incorporation of additional antigens—e.g., ROP18, GRA14—in multivalent PLGA formulations may yield synergistic protective effects and reduce escape mechanisms by parasite stage switching.

### 1.6 T-cell epitope mapping

Identification of TgGAP45-derived epitopes presented by MHC-I/MHC-II molecules in vaccinated hosts could inform the rational design of synthetic peptide vaccines and MHC tetramer development for immunomonitoring.

## 2 Conclusion

Zhou et al. have laid a robust foundation for nanotechnology-enabled toxoplasmosis vaccination, and their study stands as a benchmark for rational subunit vaccine engineering. Expanding upon their approach in the directions outlined above could yield a next-generation vaccine that is not only safe and effective but also field-deployable for both human and veterinary applications. Given the zoonotic impact and global prevalence of toxoplasmosis, this line of research is not only scientifically sound but of pressing public health importance.
